# Virus community views on the possible extension of virus taxonomic assignments to a below-species rank

**DOI:** 10.1099/jgv.0.002313

**Published:** 2026-08-19

**Authors:** Peter Simmonds, R. Curtis Hendrickson, Holly R. Hughes, Jens H. Kuhn, David L. Robertson, Sead Sabanadzovic, Arvind Varsani

**Affiliations:** 1 Centre for Virus Research, MRC-University of Glasgow, Sir Michael Stoker Building, Glasgow G61 1QH, UK; 2 Department of Microbiology, University of Alabama at Birmingham, Birmingham, AL 35294-2170, USA; 3 Centers for Disease Control and Prevention, Fort Collins, Colorado, USA; 4 Frederick, Frederick, MD 21704, USA; 5 Department of Agricultural Science and Plant Protection, Mississippi State University, Mississippi State, MS 39762, USA; 6 Institute for Genomics, Biocomputing and Biotechnology, Mississippi State University, Mississippi State, MS 39762, USA; 7 The Biodesign Center for Fundamental and Applied Microbiomics, School of Life Sciences, Center for Evolution and Medicine, Arizona State University, Tempe, AZ 85287-4701, USA

**Keywords:** International Committee on Taxonomy of Viruses, nomenclature, species, taxonomy, virus classification

## Abstract

The International Committee on Taxonomy of Viruses (ICTV) is considering a proposal submitted in July 2025 to create an optional additional taxonomic rank below the level of species. This was designed to increase the precision of taxonomic assignment where clinical or other regulatory distinctions need to be made between viruses assigned to the same species. To understand current classification practice and to gauge opinion on the proposal to formalize a below-species taxon, current ICTV study group members (*n*=746) representing over 100 Study Groups were invited to complete a survey, from whom 240 completed forms and comments were collected in November 2025. The survey also recorded opinions on the use of designators, such as ‘type’, ‘isolate’ and ‘subspecies’ for below-species assignments and the typographic format of their names, for example, whether to be italicized and/or Latinized.

Overall, a 6 : 1 majority favoured the introduction of an optional below-species taxonomic rank managed by the ICTV, and was particularly supported by those working in animal and veterinary virology fields where various forms of community-supported below-species classifications are used. There was also support to formalize and taxonomically assign existing classifications of viruses and define their nomenclature. Names might be formatted using the Latin alphabet but with less support for italicization or for Latinization of the below-species epithet. The survey data and associated comments will be considered in further discussions at the next ICTV Executive Committee meeting in November 2026. We thank all of their survey respondents for their invaluable and expert contributions to this consultative process.

## Introduction

The International Committee on Taxonomy of Viruses (ICTV) is responsible for the classification of viruses into taxa ranging from species (lowest rank) to realm (highest rank), codifying the demarcation criteria used for assignment of viruses to taxa and specifying their nomenclature [1]. The ICTV provides a comprehensive taxonomy of viruses and some related mobile genetic elements and maintains the official Master Species List (MSL) of classified viruses [2] and associated resources [3, 4]. Viruses can be classified based on the availability of coding-complete genome sequence(s) [5] without any historical requirement for the assignment of a type species [6] or deposition of type specimens or isolates into formally recognized bioarchives that is typically performed in other biological taxonomic codes.

Viruses cannot be classified based on the biological species principle where demonstration or inference of interbreeding capacity and sharing of genetic pools was used for the original assignments of animal and plant species [7, 8]. However, the taxonomic codes for viruses (and prokaryotes) do use the species rank and assign viruses to that rank based on their phenotypic descriptions and more recently, based on genetic divergence thresholds that reflect, at least in part, patterns of genetic grouping, epidemiologies and phenotypic properties, such as host range [1, 9, 10]. While the species definition for viruses specifies that members of a species must share a common evolutionary origin distinct from other known viruses (i.e. to be monophyletic) [11], the definition does not entail any pre-specified threshold of nucleotide sequence identity that demarcates species.

In prokaryotic taxonomy, degrees of genetic relatedness include average nucleotide identity with a threshold of 95% that broadly demarcates species membership based on historical and typically phenotypically described species assignments [12, 13]. In contrast, species demarcation criteria for viruses are highly variable, in part the result of inheriting traditional disease-based or other phenotypic assignments from a pre-genomic era when their genetic relationships were unknown. For instance, as described previously [14], the *NS5* gene of viruses assigned to some distinct orthoflavivirus species of the family *Flaviviridae* may differ by as little as 1.5% (e.g. Israel turkey meningoencephalomyelitis virus (*Orthoflavivirus israelense*) vs. Bagaza virus (*Orthoflavivirus bagazaense*)) to 44% pairwise distance comparisons (San Perlita virus (*Orthoflavivirus perlitaense*) and Ilheus virus (*Orthoflavivirus ilheusense*)). Similarly, viruses of different mitovirus species of the fungi-infecting virus family *Narnaviridae* are 64–82% divergent and their sequences can barely be aligned. While this is often arbitrary, it works as a functional taxonomic system as most historically-inherited species rank assignments have been stable for many decades and are in standard use by the field. However, the increasing number of viruses that have been identified and classified based on their genome sequences alone has encouraged the development of greater standardization of genetic demarcation thresholds for species and higher rank assignments. For example, species of metagenomically described bacteria-infecting viruses are now typically assigned using a metric of 95% intragenomic similarity [15].

While a variable species demarcation threshold is not intrinsically problematic in virus taxonomy generally, many important virus groups, such as human immunodeficiency virus 1 and polioviruses are not described taxonomically, forming groups below the species rank. Poliovirus types 1, 2 and 3, for example, are all classified in the species *Enterovirus coxsackiepol*, along with several other coxsackie A viruses and numbered enteroviruses [16]. Thus, regulations for containment and bioarchiving of poliovirus samples and isolates have to be framed using virus descriptions and definitions that are outside of the remit of the ICTV. Many of the large number of comparable below-species groups, assigned as clinical, veterinary or crop pathogens, recently zoonotic viruses, such as SARS-CoV-2 and those subject to specific export/import restrictions are similarly taxonomically undifferentiated from other members of the species to which they are assigned. This can create quite variable nomenclature and reference; virus lists produced by organizations, such as the World Health Organization (WHO) Priority Pathogens [17], the UK regulatory list of dangerous pathogens [18] and indeed NCBI and other INDSC databases, often use a mix of hybrid virus species and virus names for viruses, instead of standardized taxonomic assignments.

### Proposal for a below-species rank

A proposal was submitted to the ICTV Executive Committee meeting (EC57 in Birmingham, Alabama, July 2025) to expand taxonomy through the creation of an optional additional taxonomic rank below the level of species [19]. This was designed to increase the precision of taxonomic assignment in specific cases where clinical or other regulatory distinctions need to be made between viruses assigned to the same species. This proposal followed the publication of a previously published critique of virus classification and taxon nomenclature [20]. If accepted, this proposal would enable the classification of viruses to be extended to an optional subspecies rank, providing existing assignments to types, serotypes and genotypes to be re-categorized as formal taxa where this was believed to be important for clinical or regulatory purposes. The proposal was discussed at the meeting and online between EC members, with the outcome that it would be useful to obtain feedback from the wider ICTV, virology communities and other users of virus taxonomy before deciding on whether to adopt the additional rank. Accordingly, the proposal was assigned the status of Ud (U: Under consideration; d: further consideration deferred (normally to the following year)) meaning that it can be re-presented this year at the annual ICTV meeting (‘EC58’) in November 2026 for further discussions.

To summarize briefly, the proposal outlines the establishment of a single below-species rank to which viruses can be assigned, where required, and on an optional basis. Below-species names could be derived from names that are in use for viruses, such as *E. coxsackiepol* type *Poliovirus 1,* whereas other picornavirus species could stay unclassified below-species – assignments would thus be based on practical utility as they are in other taxonomic codes. Furthermore, classification of one or more viruses into below-species ranks (e.g*. Betacoronavirus pandemicum* isolate *SARS coronavirus 2*) would not require other members of the species, such as the many bat sarbecoviruses in this example, to be similarly classified.

The establishment of an optional below-species rank was argued to have advantages of clarity, accessibility, assignment histories, attributions, transparency and permanence of recording by the ICTV and the proposer believed that this approach to be preferable to what might be perceived as a largely ad hoc, often outdated and sometimes conflicting ways current community-based sub-species classifications are documented and published.

On the other hand, it could be argued that a below-species rank may increase the complexity of virus taxonomy. There might further be little benefit in classifying virus groups taxonomically when they are currently adequately defined as viruses with an accepted community-based understanding of their species definitions and their inter- and intra-species demarcation criteria.

### The survey

To gauge opinions from the wider virology community on the value and practicality of introducing a below-species rank in ICTV taxonomy, we developed a questionnaire to determine current practice with virus classification below the species rank, how information is disseminated, respondents’ attitudes towards a future taxonomic proposal that might formalize a single below-species rank and what advantages and problems might be associated with this step. There were some supplementary questions on how below-species taxonomic names might be formatted.

The survey was distributed to a large cross-section of the virus community. Specifically, we targeted a total of 746 current members within the 128 Study Groups that advise the ICTV on taxonomy, as they will have, in addition to their virological expertise, some practical experience of virus taxonomy, the ICTV taxonomic code and the writing and submission of formal taxonomy proposals. Although the survey included some members of the ICTV in their capacity as Study Group Chairs or Executive Committee members, the bulk of the 240 survey responses were obtained from Study Group members. The responses are summarized below, and specific comments are recorded in the Appendices.

## Methods

The survey was sent to a total of 746 members of ICTV Study Groups. Invitations to participate were sent out by the ICTV President, Murilo Zerbini, on the 10 November 2025, with responses to be submitted by the 25 November 2025 (2 weeks). By that deadline, 240 responses had been received and were analysed for the study.

The survey used Google Forms and can be viewed here [21].

The Introduction to the survey contained a brief introduction to the proposal, and a critique of its potential value and drawbacks along the lines of the current Introduction. Results were provided in the form of a spreadsheet (File S1) that tabulates all voting and comments on the form. A further document lists the comments received for each question in a list format (File S2). Some information has been redacted to preserve the anonymity of the participants.

## Results

**Fig. 1.**
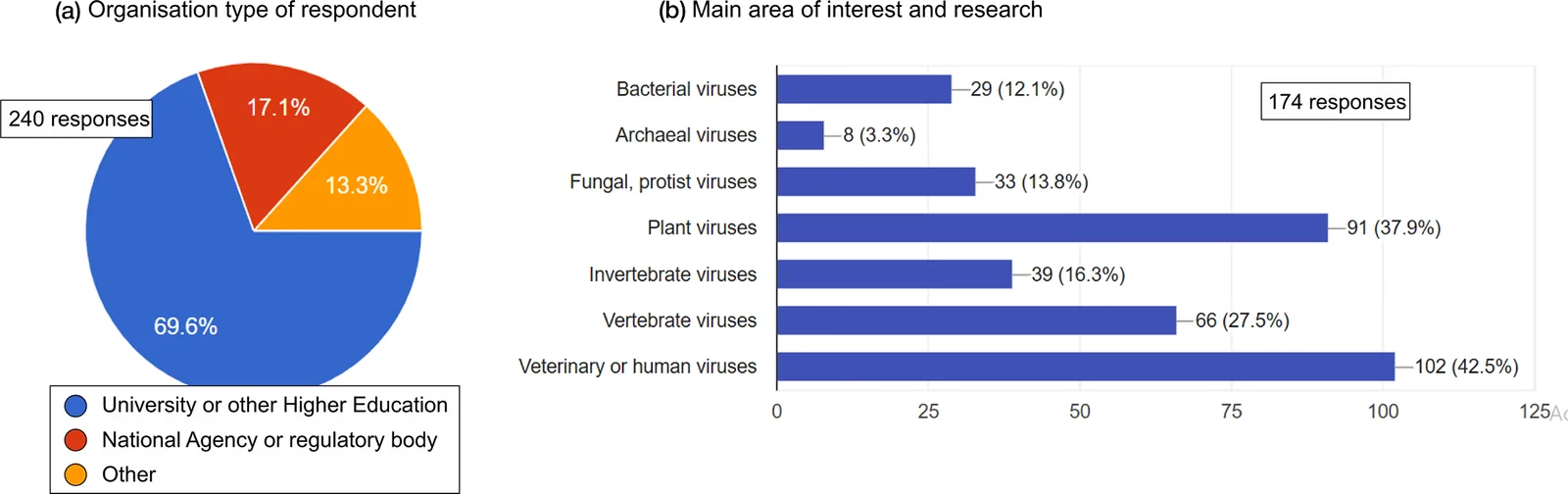
Professional background of the survey recipients, in terms of (a) affiliated organization and (b) area of virus interest and research

A total of 240 responses were received. These originated primarily from individuals associated with higher education institutions (70%), others from national agencies (17%) or named research institutes (13%) (Fig. 1a). Respondents were drawn primarily from members of Study Groups without a formal ICTV affiliation (184; 77%), Study Group Chairs (49; 24%) or other ICTV members (23; 9.6%).

There was a wide range in the viruses studied by the respondents (Fig. 1b), with substantial representation from virologists working on medical and veterinary viruses, other vertebrate viruses and plant viruses. This differentiation was useful given that the value or otherwise of a below-species rank may vary by virus group (and has been used to analyse responses below).

### Involvement in below-species classification

**Fig. 2.**
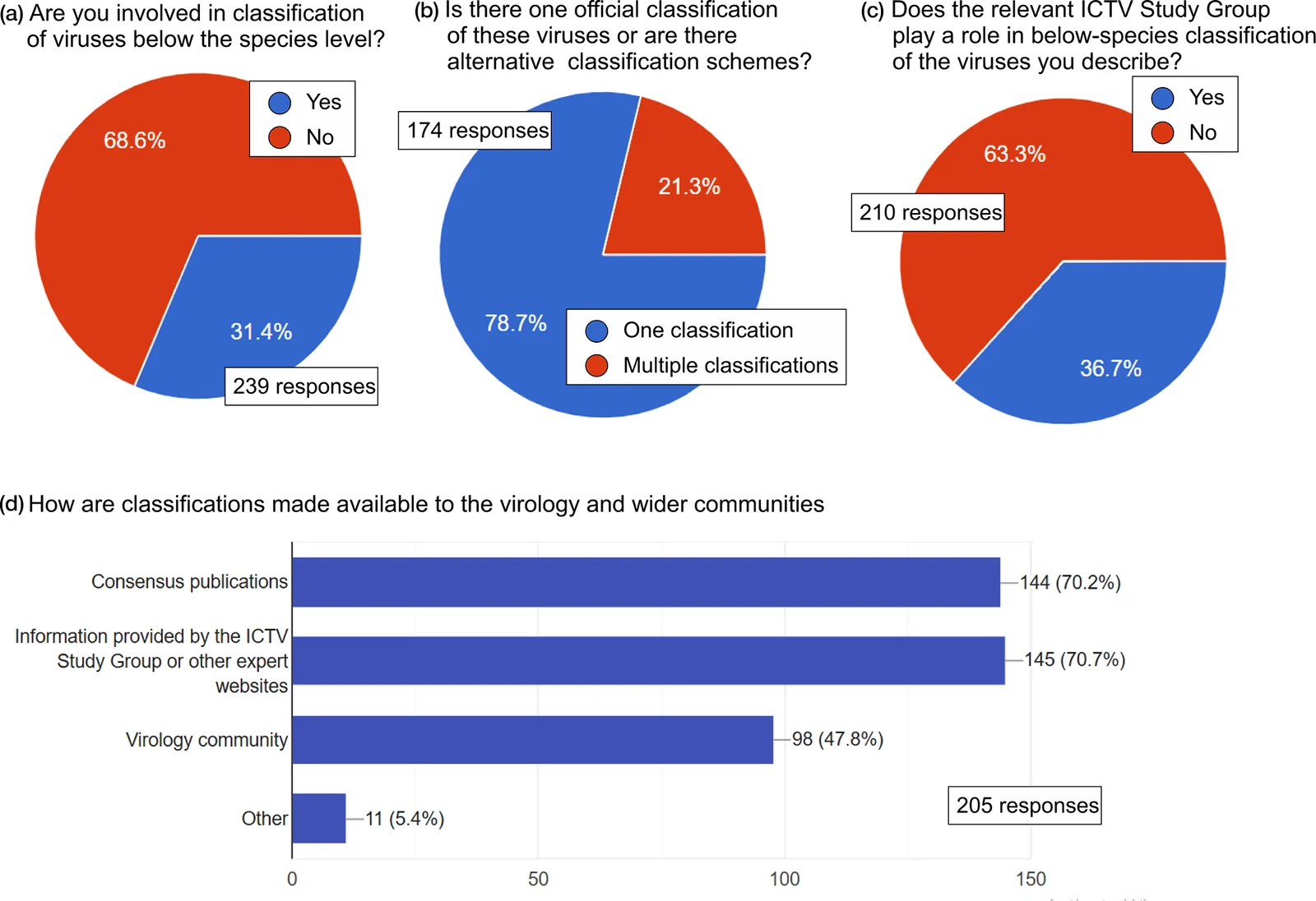
(**a**) Engagement of survey respondents in below-species classification, (**b**) the existence of defined or multiple below-species classifications used by virologists and the (**c**) involvement of the relevant ICTV Study Group in that activity (albeit formally beyond its remit). (**d**) How below-species classification information is disseminated – a list of publications and websites provided by the respondents is listed in Section B, File S2.

Around one third of respondents were actively involved in below-species classification (Fig. 2a), which entailed the publication and other dissemination of consensus (79%) or more varied (21%) classification schemes. Respondents indicated that there was usually one formally used classification of viruses below the species rank. Classifications were maintained and provided to the virus community both through the ICTV (37%), for example through the inclusion of additional isolates in the virus metadata resource [22], or through consensus publications co-authored by ICTV Study Group members. However, the large majority of below-species classifications were reported to be produced by the broader virology community outside of the remit or supervision of the ICTV. Several consensus documents in this category are listed in Section B (File S2). The responses indicate a widespread need for classification below species that is not officially provided by the ICTV, even though Study Group members may be informally involved in this activity.

### Using a formal below-species taxonomic rank

**Fig. 3.**
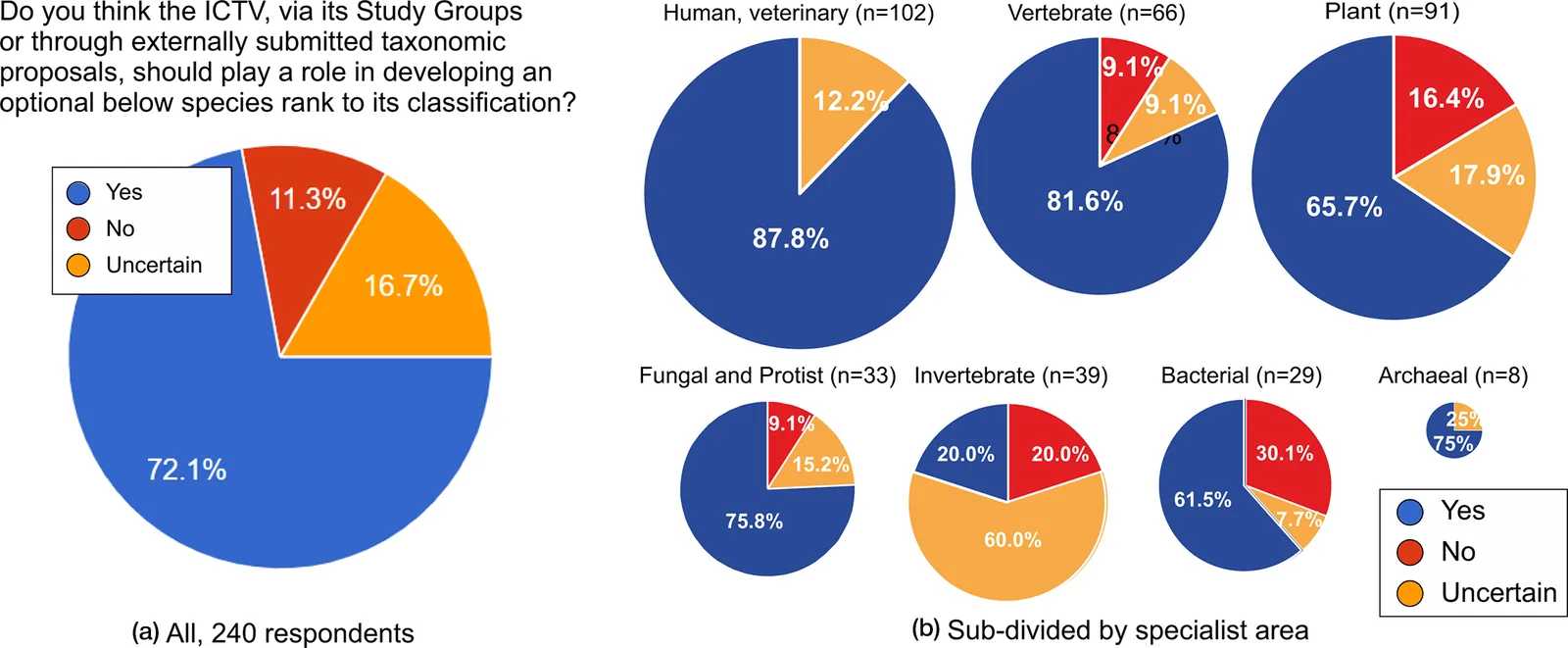
Opinions on whether the ICTV should play a formal role in the assignment of viruses at below-species level (**a**) all responses, and (**b**) subdivided by specialist area, with the pie charts scaled by numbers of respondents in each category.

The key questions asked of the respondent in this section concerned their attitudes towards a potential role of the ICTV in organizing and regulating below-species classification, and the value of such classifications for particular stakeholders (Fig. 3a).

A clear majority of respondents (72%) believed that the ICTV should be involved in the formal assignments of below-species taxa through the submission of taxonomic proposals. Although 17% of respondents expressed uncertainty about this, and excluding these, there was an approximate sixfold greater number who agreed with the proposal compared to those who disagreed.

The implications and opinions potentially vary between those working with different virus groups, as these differ from each other in the extent to which below-species classifications are currently used – classification of viruses into sero/genotypes or geographically named isolates is common among human and veterinary viruses, but are not typically used among bacterial and archaeal viruses.

To determine how professional engagement with different virus groups influenced attitudes towards support for formal below-species classification, the responses above were divided by subject area (Fig. 3b). There was proportionally greater support for below-species classification by the ICTV amongst clinical and veterinary virologists with nearly 9 out of 10 (88%) favouring the introduction of a below-species rank. For virus groups where there is little existing below-species classification, such as plant, fungal, protist, invertebrate and bacterial viruses, opinions were more varied but with a majority in all but one of the groups supporting an ICTV role in classification at a lower rank.

**Fig. 4.**
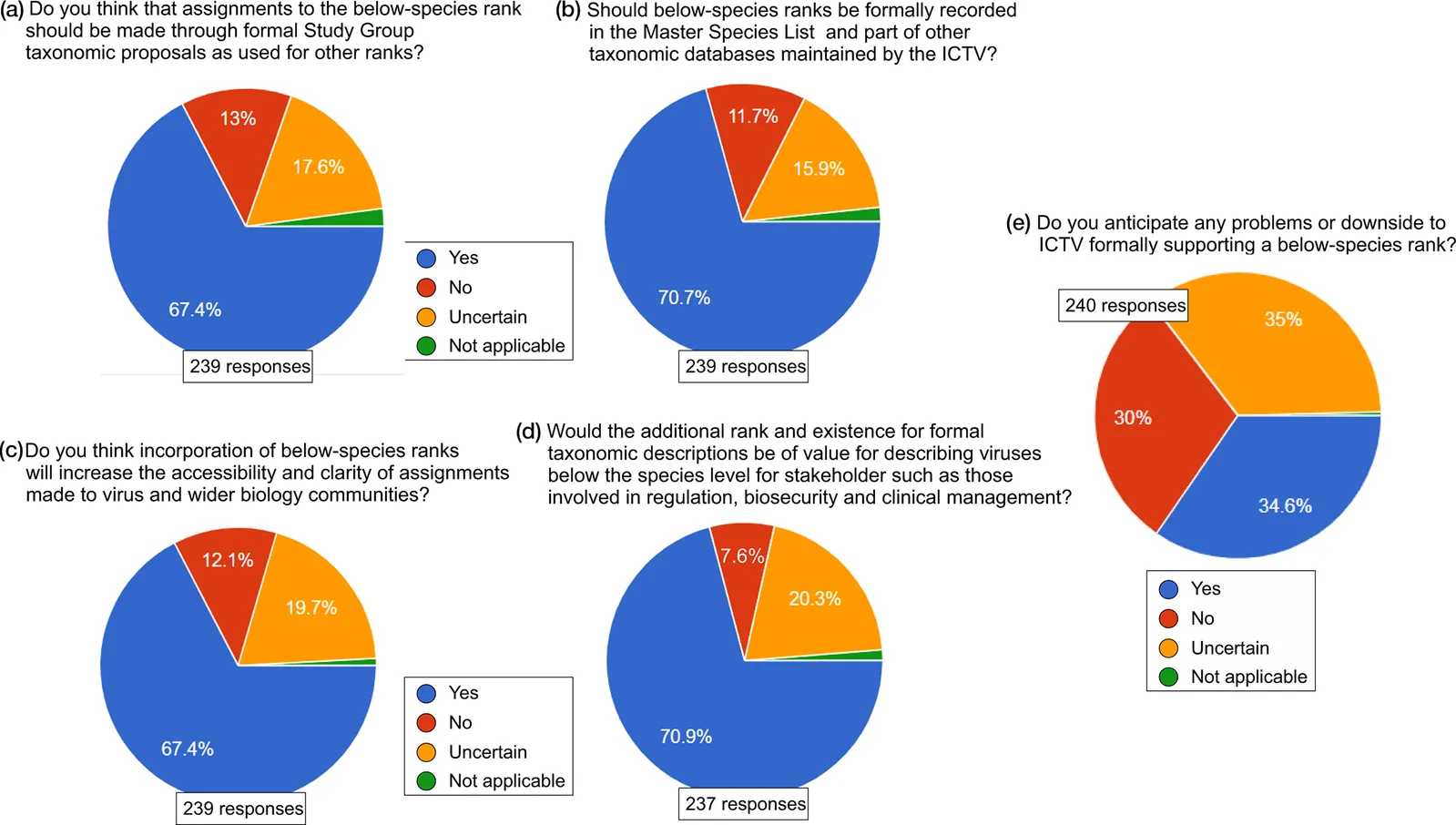
(**a-d**) Opinions of the mechanisms of introducing a below-species rank by the ICTV and the value of assignments for the wider virology community and other stakeholders. **e**) Opinions on the extent to which adding the below-species rank might create problems for the wider community – descriptions of these are listed in Section C (Supplementary Data File S2).

A series of follow-up questions reviewed how a below-species classification might be managed by the ICTV, and the extent to which current procedures for extending or modifying the virus taxonomic classification might be extended for a new rank (Fig. 4a, b). Nearly three quarters of respondents believed that taxonomic assignments might be based on the submission of standard taxonomy proposals via the relevant ICTV Study Group and Subcommittee. It was generally believed that taxon assignments and other classification information for below-species taxa should be recorded in the MSL and other taxonomic databases. These views support the idea of a relatively seamless expansion of ICTV taxonomy to a below-species rank managed by the same Study Groups that are responsible for species and higher rank assignments. Similar proportions of respondents believed that assignments would improve accessibility of that classification information to the wider community and its potential value to stakeholders. Responses were broadly similar to the proportions who supported an ICTV role in this process (see above), with a ratio of around 6 : 1 of those in favour of the proposal (excluding ‘Uncertain’).

Respondents were split in their opinions over whether the additional rank may create problems and have other potential downsides, with around a third voting in the Yes, No and Uncertain categories. Expressed concerns ranged from the practicality of organizing an additional rank, questioning whether the ICTV should play a role in below-species classification when there might be more effective and representative community regulation, and the justification for the rank if taxa are not to be classified equivalently to species and higher ranks. However, many of the comments were predicated by an (incorrect) assumption that variants within every species should be classified at a below-species rank, rather than this being an optional rank, as subspecies are in other taxonomic codes.

### Format of below-species nomenclature

If such a below-species rank was to be introduced, there would have to be clear guidelines on how its taxa should be named and formatted. As with discussions over the format of species names in previous years, several questions were asked concerning allowable characters, italicization and Latinization and the extent to which names of taxa at below-species ranks should follow existing virus names as much as possible:

To briefly summarize the responses, there was strong support to formalize established nomenclature of separately named viruses or potentially of assigned types for below-species taxon names. The majority of respondents believed these should be written in normal type but with a similar restriction on special characters as imposed on species names. There was only around 20% support for rank names to be italicized despite this formatting being used at all current virus taxonomic ranks. There was strong disagreement with Latinization, reflecting at least in part the idea to preserve existing virus names as much as possible.

**Fig. 5.**
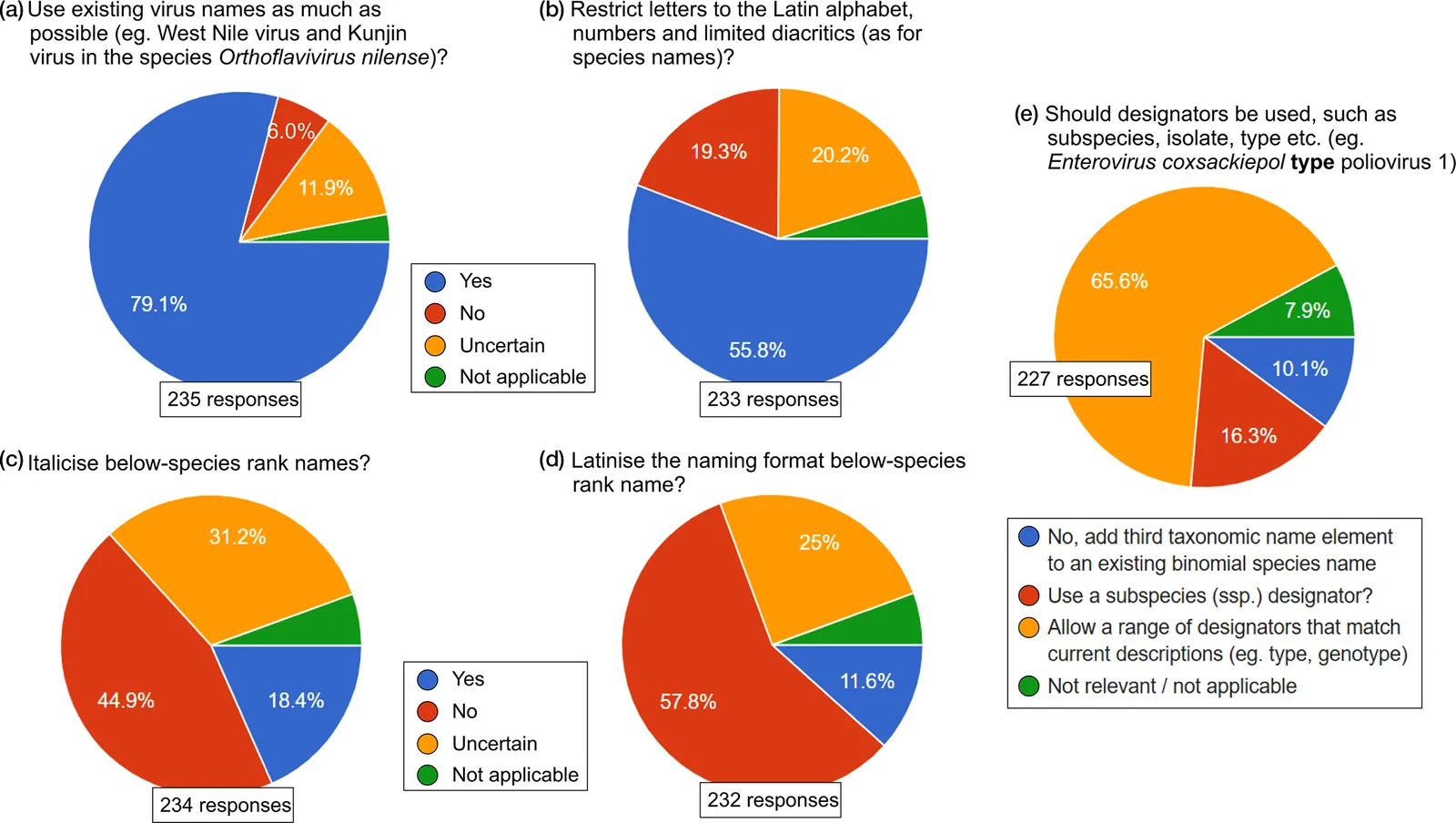
(a-d) Opinions on the naming rules and typographic formatting of below-species names species should they be introduced. (**e**)

In keeping with minimizing disruption of established nomenclature, proposal 20215.001G also suggested that ‘designators’ might be used for below-species taxon names, somewhat analogous to their use in botanical below-species names, such as ‘cultivar’ and ‘variety’. Designators might include ‘type’, ‘serotype’, ‘isolate,’ etc. This proposal was broadly supported in the survey responses (Fig. 5e).

There were some general comments on nomenclature, primarily concerning the relationship between virus and what would be taxon names, and also comments about the lack of standardization of existing below-species names that may favour the use of designators over subspecies. Again, this may reflect the need to preserve elements of existing classifications and virus names in some form.

## Discussion

The ICTV has been uniquely mandated by the International Union of Microbiological Societies (IUMS), formerly the International Association of Microbiological Societies (IAMS), to develop and maintain the taxonomic classification of viruses since 1966. Over its period of existence, it has successfully developed a comprehensive and universal taxonomy of viruses and some related mobile genetic elements through the efforts of several generations of volunteers drawn from the virology and wider microbiology communities. The ICTV is managed by a rotating Executive Committee and appointed Study Group Chairs, and currently engages with over 700 virologists drawn from the wider community to provide expertise in taxonomy discussions and proposals for the many different virus groups. It participates in wider outreach activities, including the provision of comprehensive database resources on the ICTV website [23] and the publication of the online (tenth) report [24].

It is in this spirit that the ICTV seeks to consult with the wider community on potential changes to taxonomic practice. This has included the large-scale consultation on the standardization of a binomial name format for virus species, using a typography of genus +species epithet used in scientific names of other taxonomic codes [25]. Given the extensive community usage and regulatory importance of the nomenclature of viruses classified into groups beyond the species rank, the ICTV Executive Committee has decided that a community consultation on the proposal to create a below-species rank for viruses classified at this level would be valuable: first, to understand how classification is currently performed; second, to gauge the involvement of virologists affiliated and non-affiliated to the ICTV in this task; and third, to seek community views on how a classification might be established within the auspices of the ICTV and how taxa at this rank might be named.

The survey findings demonstrate that an overwhelming majority of the 240 respondents favoured the extension of the formal virus taxonomy to include below-species groups by the ICTV, albeit with less clarity on how to accomplish that. This step would provide virus taxonomy with extended classifications that are analogous to subspecies used in other taxonomic codes for animals, plants, fungi and prokaryotes. These maintain one or more (in the case of some plants) optional below-species ranks to enable classification of variants of organisms within a species. For bacteria, subspecies may be assigned based on their distinct clinical or other phenotypic properties [26], whereas animal and plant subspecies tend to be assigned to geographically separate populations that have come to be phenotypically distinct even though they retain an interbreeding capability [27, 28]. For viruses, the principal reasons for establishing a below-species classification revolve around distinct clinical presentations and epidemiologies or epizootiologies of their infections, host range changes in zoonotic infections and in regulatory differences, such as in handling requirements of samples or virus cultures at different biocontainment levels.

The proposal to create a below-species rank will be discussed again at the next Executive Committee meeting in November, 2026 where the polled opinions and detailed comments on practicality, scope and nomenclature of below-species groups will contribute to the discussion and final decision. The poll will remain open and readers may wish to contribute further opinions and comments in advance of the meeting here [21]. We thank the generous contribution of time and thought given by the many respondents to this community poll and hope that any taxonomy changes arising from the proposal will address the broader aims and concerns of the wider virology community.

## Supplementary material

10.1099/jgv.0.002313 Supplementary Material 1.

10.1099/jgv.0.002313 Supplementary Material 2.

## Abbreviations

ANI: average nucleotide identity
ICTV: International Committee on Taxonomy of Viruses
MSL: Master Species List
