# Supplementary material for: Virus community views on the possible extension of virus taxonomic assignments to a below-species rank
Source: J Gen Virol. 2026 Aug 19;107(8):002313. doi: 10.1099/jgv.0.002313 (PMC13489588; doi:10.1099/jgv.0.002313)
Supplement: Supplementary file 1 [file jgv-107-02313-s002.pdf]

Suppl. Data File S2.

Comments and responses to specific survey questions:

B) Current Practice

1) *Are you involved in classification of viruses below the species level? List examples of these and / or provide classifications/resources/publications links (URLs) associated with these (71 responses)*

- Papillomavirus and polyomavirus types
- Oliver SL, Dastjerdi A, Wong S, El-Attar L, Gallimore C, Brown DW, Green J, Bridger JC. Molecular characterization of bovine enteric caliciviruses: a distinct third genogroup of noroviruses (Norwalk-like viruses) unlikely to be of risk to humans. J Virol. 2003 Feb;77(4):2789-98. doi: 10.1128/jvi.77.4.2789-2798.2003.
- Dastjerdi A, Benfield C, Everest D, Stidworthy MF, Zell R. Novel enteric viruses in fatal enteritis of grey squirrels. J Gen Virol. 2020 Jul;101(7):746-750. doi: 10.1099/jgv.0.001431.
- rotavirus classification working group
- SARS-CoV-2, most prominently, but the issue comes up in every committee decision to some extent. https://www.nature.com/articles/s41564-020-0695-z
- PepMV (Potexvirus pepini) classification into genotypes. Review in preparation
- Kropinski et al. classifications for actinophages
- rice yellow mottle virus; sobemovirues
- hantaviruses, coronaviruses, flaviviruses
- https://pubmed.ncbi.nlm.nih.gov/40609048/ ; https://pubmed.ncbi.nlm.nih.gov/37561168/
- CCHFV, AAVs, FMDV, SARS-CoV-2
- Types in for benyviruses
- Just an example of plant virus strains classifications that we are working with sometimes: this is for PVY strains: https://doi.org/10.1094/PHYTO-10-10-0284
- https://www.mdpi.com/1999-4915/12/11/1240
- Filoviridae family Study Group
- genotype and subtype assignment tool development for various human and animal viruses
- Porcine circoviruses
- BTV classified below species level based on constellation of segments (PMID: 26252219)
- https://www.picornaviridae.com/
- As member of the ENPEN steering/scientific committee and molecular virologst, I am involved in classification of possible new genogroup, clades, lineages of enteroviruses, particularly when these arise during an outbreak. These are the unofficially classified through a publication
- Avian metapneumoviruses subtypes A, B, C or D, with A,B,D being more similar to each other than C, which is more related to Human Metapneumovirus (ref Brown et al, DOI: 10.1371/journal.pone.0102740)
- https://pmc.ncbi.nlm.nih.gov/articles/PMC6950264/; https://pmc.ncbi.nlm.nih.gov/articles/PMC8880664/
- https://doi.org/10.1099/jgv.0.000519

- https://doi.org/10.1016/j.vetmic.2016.02.008
- https://doi.org/10.1016/j.vetmic.2011.11.003
- https://doi.org/10.1637/10323-081012-resnote.1
- https://doi.org/10.1099/vir.0.057711-0
- https://doi.org/10.1016/j.vetmic.2013.05.017
- Tick-borne encephalitis virus, Louping ill virus, DOI: 10.3390/v17010122
- Infectious salmon anaemia virus (ISAV) genotypes; Piscine orthoreovirus (PRV) genotypes
- Coronaviridae ICTV Report author, Anelloviridae ICTV Report author
- Orchid fleck virus strains and variants
- https://www.who.int/news-room/fact-sheets/detail/mpox
- RVA: https://pubmed.ncbi.nlm.nih.gov/41173130/
- RSV: https://pubmed.ncbi.nlm.nih.gov/40096248/
- MPXV: https://pubmed.ncbi.nlm.nih.gov/38639093/
- ICV: https://pubmed.ncbi.nlm.nih.gov/37536021/
- HAdV: https://pubmed.ncbi.nlm.nih.gov/31817764/
- Classification of norovirus genogroups, genotypes and P-types.
- Strains of plum pox virus
- Family Papillomaviridae; Genus Alpha-PV; Species Alpha-9; Type HPV16; Variant HPV16-A
- Circoviruses
- Rotavirus Classification Working Group (RCWG): https://rega.kuleuven.be/cev/viralmetagenomics/virus-classification/rcwg
- Hepatitis C Virus genotypes, but this genotyping happened before I joined the study group
- Noroviruses are another example of necessary subspecies classification, here I am not in the study group.
- Use published literature as reference, for serogroups often refer to older resources. I manage to Arbovirus Reference Collection at CDC, we still use the original virus names assigned when isolated. We update the family, genus, etc. if a virus is reclassified, but we don't change virus names except in rare cases.
- Subtypes of Venezuelan equine encephalitis virus, which impact select agent designations in the U.S.
- Genotypes/biotypes of turnip mosaic virus, and of Alternanthera mosaic virus
- picornaviruses and hepatitis E viruses
- Separation of genotypes to clades for epidemiology e.g. https://apsjournals.apsnet.org/doi/full/10.1094/PHYTOFR-05-24-0049-A but also use of biology to determine strains which can have relevance for certification or regulation . e.g. https://link.springer.com/article/10.1007/s00705-019-04395-7
- https://rega.kuleuven.be/cev/viralmetagenomics/virus-classification/rcwg

2) *Are you involved in classification of viruses below the species level? List examples of these and / or provide classifications/resources/publications links (URLs) associated with these (52 responses)*

- There is not an official ICTV classification system Plant virologist follow. In general the scientific community use biological differences in virus expressions in differential plant species (mostly herbaceous, but not limited, that include monocots and dicots). These difference are also associated to detectable differences in mechanical transmission

efficiencies and transmission by arthropod vectors. Also, overcoming the resistance in knowingly resistant varieties.

- I fully support the proposal. If some virus group has the below species level of their classification, which is commonly used in practice, its official approval will make virus classification much easier and practically applicable.
- Current genotype classification of hepatitis C virus and hepatitis E virus is a product of the ICTV Flaviviridae and Hepeviridae Study Groups
- Previous nomenclature allowed the addition of -Sin to denote the isolate.
- SARS-CoV-2...
- The phage community is still using the Myo, Podo, Sipho descriptions and without direct linkages to the new names it's a really confusing time in taxonomy right now.
- no
- For the virus families I work with (Endornaviridae and Hadakaviridae), there is currently no established practice of below-species classification. Endornaviruses lack infectivity and cannot be experimentally transmitted, making it impossible to assess biological properties such as host range, pathogenicity, or serological relationships that typically form the basis for below-species classifications in other virus groups. Species demarcation in these families is based primarily on sequence similarity and phylogenetic relationships, and the current species-level classification provides sufficient resolution for our research and communication needs.
- I do not quite understand the above question. Is not that of ICTV the only offical classification? What does "these viruses" refer to? I work with human parvoviruses, and the below-species parvovirus names are quite established.
- Example: CPV-2/FPV (Protoparvovirus carnivoran1) classification is pretty clear (the 2 viruses are distinguished by several aa differences and host tropism). However, classifications based on types and geographical origin, not phylogenetically supported, exist and are a controversial matter. In this case (and I am sure that there will be others), it would be handy to provide a unique reference framework.
- in baculoviruses isolates from different host species are usually named using the host species by the researchers, even if the taxonomic and phylogenetic relevance is not useful. This might however indicate some adaptive specialisation.
- Types are named by name of host and are numbered consequtively (human adenovirus 41, raptor adenovirus 1). However, different authors describe the same virus by the name of the host they just discovered or by very general names (amniote adenovirus 1, passerine adenovirus 1) or just by isolate numbers which may refer to very similar viruses (variants).
- For hantaviruses, there is yet no additional sub-species classification. However, within one species, e.g. Orthohantavirus andesense, different levels of pathogenicity have been suggested, depending on the geographic context (e.g. Andes virus South, Andes virus Nord, etc.). However, in many cases we still miss whole genome sequences to establish their classification: https://pubmed.ncbi.nlm.nih.gov/36992369/
- There are multiple proposed genotyping systems for ASFV, while for ISVs these are limited and less well defined, particularly for segmented viruses.
- There is a proposed classification of human respiratory virus below the species level (Emerg. Infect. Dis. 2021;27:1–90. A follow up proposed classification below the subgroup level has been published in 2024 (Emerg Infect Dis. 2024;30(8):1631-1641).
- We have found the sub-species classifications helpful with respect to tracking the growth/decline of lineages over time and their movements around Africa.
- Important to have flexibility to have below species classification at least in medical microbiology and when we are working on genomics evolution ....

- For plant viruses, below species classification is complex, for example in potato virus Y: considering potato isolates, this virus is usually classified in strains, while for pepper isolates, they are classified in pathotypes.
- In the preceding question, I have to read "classification" as "nomenclature". In my field, a species contains a single virus that exists as many strains or variants. The relevant nomenclature for viruses and strains is assigned and modulated by the field. Hence, for example, a virus may historically have more than one name.
- Papillomavirus type classification is very structured through the International Human Papillomavirus Reference Center or the Animal Papillomavirus Reference Center. Polyomavirus type classification is less formal but largely consistent.
- As a member of the Arenavirus Study Group by using morphotaxonomy, genetics, eco-pidemyiolgy and all the tools to manipulate the data there is no need for an infra-species formal classification.
- I'd argue the silly WHO and popular variant names are not really official, but they are treated as such by the media and the public.
- In general terms, the classification in the latest review is accepted and used
- https://journals.asm.org/doi/10.1128/jvi.01234-19; reclassification of Ross River virus into 4 distinct genotypes
- I think SARS-CoV-2 and seasonal influenza A viruses are good examples of how consensus-driven classifications—combining phylogenetics and antigenic cartography—have defined sub-species groupings. I agree with the premise of the new proposal to make these sub-species classifications more systematic to bring greater clarity to the literature, and I would be happy to contribute to any future efforts.
- I am part of the ICTV Herpesvirales Study Group. We do not yet have or use below-species classifications for the human herpesviruses that I study, but I can foresee that this may be a useful option in the future. (e.g., VZV has well-accepted clades below the species level, and incorporating these into a formal sub-species categorization could be useful.)
- By only genome sequences, it is difficult to classify papaya ringspot virus to P type or W type.
- for some of the viruses there is one (FMDV), for others there are multiple (CCHFV). Where would lineages/VOCs of SARS-CoV-2 fit into this? SARS-CoV-2 would be the sub-species element - but where is the line drawn? there? or further down into lineages of a virus which itself is a variant of an official species.
- The ICTV guidelines are followed for archaeal viruses
- one classification is the ideal but not always reality
- I think it's good to have general solid almost never changing mandatory one classification; if a group of viruses needs sub-species level - let's have it, but let's not enforce it (especially in groups where there is 1 virus in whole genera etc - these new groups of viruses)
- Ideally to homogenize existing classifications.
- it took some time tio convince the community but now it seems to work quite well, despite minor differences on single sero-/genotyoes.
- I strongly support below species classification. In case of tick-borne encephalitis virus subspecies are mentioned in many publications, and this is very helpful and should be officially approved.
- For example, PRV genotypes I and II, a and b, are also classified as PRV-1, PRV-2, and PRV-3

- classification of alphaviruses is more or less logical. classification of taxons lower than species is confusing for coronaviruses (I have some experience with merbecoviruses) is very confusing and any clarification is most needed
- General interest in viruses and especially interactions of RNA viruses with genomes of all organisms
- Alternative classification schemes for the Coronaviridae; for the Anelloviridae there is one classificaton scheme
- Classification of Caulimoviridae family is quite clear.
- Subspecies are identified as isolates or variants
- Paramyxoviruses
- RSV subgroups/lineages have recently being codified by excellent by Dr. Goya after a long history of rival nomenclatures and provide a model whereby standardisation could be accomplished:
- https://pubmed.ncbi.nlm.nih.gov/39043393/
- norovirus gengroups, genotypes and P-types
- For plant viruses, there are widely used informal below-species groupings, such as strains or genotypes. In my work, these include PVY-N, PVY-NTN, PVY-O for Potato virus Y, and CMV strains such as CMV-Fny. These systems are used routinely in the plant virology community
- For HCV it is well defined, due to Peter Simmonds work. For Noroviruses, rather multiple classifications exist (genogroups based on capsid most common, alternative on polymerase also used in emerging strains).
- It is not really well defined. We tend to talk about "isolates" or sometimes "strains"
- Historically there was an antigenic group for classification that has been moved away from as molecular has been used predominantly for characterization. We no longer use that nomenclature. We have switched to more of a phylogeny. However, we still keep the traditional virus names, to avoid confusion.
- To a large extent, genotypes differentiated by phylogeny correlate with host ranges
- In the regulatory sphere, plant virologists work on multiple different viruses, and each has different classification systems, with different biological characteristics to define known strain types, and often these are not directly linked to a genome-similarity based classification system as employed by the ICTV.
- species name and then virus vernacular name and name of isolates
- Lefavirales, currently limited to species. Sub-species options not considered.

3) *Other means to disseminate information of below-species classification (12 responses):*

- Web pages
- WHO
- Classifications can define whether an agent is reportable and, if detected, what level of response occurs. For notifiable animal diseases, response is often legislated as part of biosecurity acts etc at a jurisdictional level. Classifications are also important for defining the strain or lineage of virus for diagnostics and research purposes (eg HPAI 2.3.4.4b).
- Discussion within the associated community
- Genome databases
- Some resources available such as FMDbase, Pangolin SARS-CoV-2, Nextclade
- Presentations at conferences
- WHO committee

- other expert website (https://rega.kuleuven.be/cev/viralmetagenomics/virus-classification/rcwg)
- When we provide viruses we provide product inserts. We provide this information in the insert if it's available.
- Classification of Rotavirus alphagastroenteritidis (Rotavirus A) below the species level for all 11 gene segments is organized by the Rotavirus Classification Working Group (RCWG)

4) *Does the relevant ICTV Study Group play a role in below-species classification of the viruses you describe? (44 responses)*

- To me, as it is proposed, the below-species classification applies more to viruses rather than species
- I believe that the species criterion should be based solely on specific evolutionary patterns. Classification below the species level can take into account not only phylogenetic data but also the ecological and pathogenetic characteristics of virus groups. For example, according to phylogenetic analysis, three/seven tick-borne encephalitis virus subtypes clearly belong to the same species as the Louping ill virus group and, possibly, the Omsk hemorrhagic fever virus. A strict subspecies definition could more clearly delineate the pathogenetic characteristics of these viruses. As is evident from the example of orthoflaviviruses with segmented genomes, etc., with the increasing number of new viruses, we will face the problemt hat there will be large differences between the evolution of genes encoding proteins of the replication complex and capsid proteins. In this case, at least initially, assuming a taxonomy based on polymerase genes, subtypes could explain differences in capsid proteins.
- For plant viruses I think it's of no substantial usefulness for lower classification
- Wider community has adopted Study Group-led classification even where the decisions made were contentious/nuanced.
- I have not had the opportunity to be actively involved, so my chosen option may not be appropriate.
- I do not know
- It would be very interesting and useful if those classifications that are agreed upon, published, and accepted globally or by international organizations such as the WHO could be reported on the ICTV
- The ICTV Study Groups for Endornaviridae and Hadakaviridae focus on species-level classification. There has been no need or discussion about establishing below-species classifications for these families.
- Many researchers, even virologists, mix species and virus names, so this approach is very welcome, thank you for the initiative!
- I am not a specialist in legislation, so this comment could be irrelevant. In the case of baculoviruses that are used as biological control agents, subspecies classification could be counterproductive for product registration but useful to manufacturer to ensure quality control of the agent.
- sequence and phylogenetic analysis in definition and characterization of strains (Closteroviridae SG mainly in my personal involvement)
- Officially not, as below species classification was not our task. However, each member uses or creates such type numbers/names for their described new adenoviruses. On the other hand, there was an overheated discussion if recombinant human adenoviruses should get new (further) type/genotype numbers. The adenovirus community is still

split in this question as there are no rigid criteria how much difference should give a right to name it as a new type. It would be a hunting task to propose a valid and obligatory type designation for several hundreds adenovirus genotypes.

- Not for ASFV. I'm not aware of activities for other viruses
- For marseillviridae, our study group provide recommendations
- When a new genus is created, the Geminiviridae Study Group usually determines a species demarcation threshold and a strain demarcation threshold
- In my field, there is no need to apply additional formal taxonomy to distinguish among viruses in the same species by virtue of biological or pathogenic properties. I recognise that such a need may exist in other fields.
- Note that my responses for the above are based on if the study group would be allowed to play a role in sub species classification, then they would do that in my opinion.
- If classification involves demarcation, it is provided in few cases: historical antigenic-based may be among most reliable and important practically.
- Current guidance is on species level, though discussions at this interface are detailed
- Rules below-species classification of the viruses need be discussed inmdependly by each group and have a specific approach (rules) for each virus family. NO general rules for this below-species classification of the viruses
- Only in designating the "2" in SARS-CoV-2. The rest comes from a variety of outside-ICTV sources.
- Not yet, but traditionally, viruses have been previously designated as strains of a species. For example, "Chlorovirus conductrix" is the current species designation for multiple viruses that were previously defined as "Paramecium bursaria Chlorella virus 1", or A11 AL1A, Al2A... etc. No below species classification is formally applied at this time.
- We do not give an official taxonomic classification of below-species deviation; deviant serological reactions etc. are noted as "strains" or different "isolates" of a virus - there is no added benefit of below-species taxonomy
- Agree it would be very helpful to apply sub-species classification of genotypically and phenotypically diverse variants of, in our case, orthoflavivirus and alphavirus species
- See above comment.
- I am against the ICTV study group determining subspecies demarcation criteria in general. This responsibility should lie with each study group, depending on whether it is necessary or not. In my study group, this rule cannot be applied to our classification due to the lack of biological characteristics in many cases.
- Below species classification has not been a point of much discussion within our studygroup
- I am sure many of the people are involved, but the classification system is not provided by ICTV
- Not that I am aware of, although this could be included in the future if needed.
- below-species classification should be a matter of the study group but there should be some general guidlines come from ICTV - to not make wild west in each sub-species taxonomy
- I agree with sub-species naming, but also Please include "common names" (or actual virus name, in addition to species name) for viruses in the Master Species List. e.g. poliovirus. Trying to look up classification of a known virus is quite difficult if one doesn't know the Latin binomial, which I'm sure is the case for virologists who are not ICTV specialists. (Most virologists). Thanks!
- At this point the ICTV Study Group that I'm involved in does not play a role in below-species classification, but this is something that might prove useful in the future to

describe bacterial viruses where below-species taxonomic ranks need to be more standardised.

- At least in the kitaviridae study group we did not mention the cut off value of nucleotide and amino acid identity for Citrus leprosis virus C2 strain differentiation
- The concept of below-species does not seem necessary to me for this family of viruses.
- WHO workgroup may be inappropriate for these designations that may fit better into ICTV purview
- Our SG does not classify viruses below species
- I assume so in the past for HCV, but since I have joined, the main focus was on the binomial names and the higher oder structure of families and orders.
- I guess I worry that this might be too fine and things may then be missed with respect to those viruses with medical implications (not my area I would point out). I guess I wonder if strain/isolate designations are enough...
- I would say that currently ICTV doesn't really play a role in below species nomenclature, but probably should. There are a lot of publications and if there was one resource it would be much less confusing and more accurate. I do think we need to keep the original virus names, changing yellow fever virus to the new species name will only cause confusion. It makes it more difficult to perform literature searches if people are using different language than historically used.
- The togaviridae study group certainly has the expertise to address below-species taxa
- I see value in ICTV adding below-species classification where information is available and of interest to more than a few specialists
- As the ICTV has largely focused on sequenced based delineation, however, in plant virology, the biological variability (host range, impact) is a key factor for regulation.
- I believe that names are simply define by the group that identifies a new isolate, usually including origin (town, county, country...and numbers)
- It may be an important consideration for the group to explore this matter. There are instances where sub-species classification may have value, particularly in the Betanudivirus genus.

C) Using a formal below-species taxonomic rank:

1) *What issues do you anticipate with a below-species classification, and how would you prevent or resolve them (103 responses)?*

- The species represents the fundamental unit of taxonomy. Just as bacterial serotypes, pathovars, biovars, coliform groups etc. are scientifically and clinically relevant but remain outside formal taxonomy, below-species categories in viruses serve important practical and regulatory purposes but should not be part of the official ICTV hierarchy. Below-species ranks are defined according to practical needs; however, these categories do not represent evolutionary units (lineages) but rather technical or epidemiological groupings, which would disrupt the hierarchical structure of taxonomy. Moreover, below-species categories are difficult to define due to numerous transitional forms, and any attempt at their official standardization would likely create confusion and undermine taxonomic consistency. Therefore, they may exist and be used in practice, but they should not be formally defined or regulated by the ICTV.
- More unwanted confusion among virologists and loss of credibility for the ICTV, The community has not metabolized the distinction between virus species and virus. I would solve this issue by NOT adopting a below species classification.
- Plant viruses exist in populations; more often, what we call a virus strain is a mix of virus variants. It has been demonstrated by mechanical inoculation passages across different hosts and by studying sequences before and after insect transmission. Also, in early experiments that used SSCP (single strand conformation polymorphisms) and more recently HRM (High Resolution Melting) patterns.
- More administrative work...
- There are concerns that the increased workload resulting from the collection and verification of accurate information may become burdensome.
- Inconsistency of demarcation criteria between different virus groups/ICTV subcommittees, pollution of classification by arbitrary non-phylogenetic antropocentric features
- I certainly hope there is no problem. But different virologists may use below-species ranks in different ways and it may be a little confusing to organize these data in an orderly fashion. The involvement of the SG and the broad virology community should prevent and resolve them.
- Complicate public awareness about emerging pathogens
- It is already complex enough trying to assign viruses to species level, adding an additional level will complicate things further, especially as this will likely look different for every group of viruses because the species cut-offs already vary. This also begs the question, where does the splitting into groups stop.
- Clinicians, veterinarians, and specialists unfamiliar with the issues of virus taxonomy and evolution may be dissatisfied with the rapidly changing criteria and names. When redefining species, families, and so on, I believe it would be good to retain the familiar names for subspecies.
- Difficulty to establish criteria for the definition of these groups (molecular criteria: problems with recombinants; biological or serological criteria: viruses can evolve and change categories)
- There is already enough work with classification levels now.
- A whole new series of controversies that are best resolved within specialties rather than by the EC and ICTV vote.

- The creation of a lower-rank species inherently invites further subdivision within that category, initiating a perpetual cycle of taxonomic refinement that contributes more to conceptual ambiguity and redundancy than to meaningful distinction
- For some begomoviruses there are so many isolates that probably each researcher will like to have their "own" below species classification.
- This will take time to use a same taxonomy. Also, there could be new, emerging lineages and the update should be quick.
- SARS-CoV-2 example... speed will be essential to not be 'overtaken' by anyone who feels like naming a new virus (nCOV-19 is a great example, but also the chaos that erupted when the naming of all subsequent variants and subvariants got started). Not sure it is realistic that ICTV could prevent this from happening the next time; just too many stakeholders and too little time...?
- With ICTV designations, I always worry about timing. People tend to give the isolate/strain a name with the publication, so if that can be incorporated as a below-species rank, that's ok. But if ICTV is going to try to change it, it will further complicate a muddy issue.
- If approved hundred of phage strains would have to be officially classified
- I believe that any classifications below the species level that the ICTV supports should be those agreed upon by experts and published, as well as supported by international institutions such as the WHO. This would avoid conflicts and be very helpful when selecting the classification to be used for genomic surveillance. Examples of such consensus classifications include RSV (doi: 10.3201/eid3008.240209), SARS-CoV-2 (doi: 10.1038/s41564-020-0770-5), dengue (DOI: 10.1371/journal.pbio.3002834), etc.
- Consensus agreement on how to determine below species classification criteria , particularly in instances where viral recombination has occurred
- Given that species epithets already often include virus names, it will become even more confusing to distinguish species from "viruses". What is the position of the below-species rank category: is it a "virus" or a "sub-clade"? This highlights the fundamental flaw in the notion/definition of a "virus", which is necessarily a clade (related group) if it's not a single virion (one particle).
- I anticipate several fundamental issues:
  - Confusion about ICTV's mandate: The proposal risks blurring the distinction between classifying taxa (virus species) and naming individual viruses or viral entities. Many examples in the proposal (HIV-1, SARS-CoV-2, poliovirus types) appear to address virus nomenclature rather than taxonomic classification of species. This extends ICTV's role beyond its traditional scope and could create confusion about the organization's responsibilities.
  - Timing concerns: The virology community is still adapting to binomial nomenclature. In plant virology, many researchers continue using traditional virus names because there are few practical situations requiring taxonomic species names rather than virus entity names. Adding another taxonomic layer at this stage may increase confusion rather than clarity.
  - Uneven utility across virus groups: The need for below-species classification varies dramatically. For families like Endornaviridae and Hadakaviridae, where viruses lack infectivity and classification is sequence-based, there is no practical need. This could lead to inconsistent implementation across Study Groups.
- To address these issues if the proposal proceeds:
  - The distinction between taxonomic classification (ICTV's role) and virus naming (community's role) should be clearly maintained

- Implementation should remain truly optional with no expectation that all species require subdivision
    - Clear criteria should be established for when below-species classification is appropriate
    - Priority should be given to reviewing existing species demarcation criteria before adding new ranks
- Extra work for ICTV volunteers. Below-species classifications will be useful only if there are clearly consistent phenotypes associated with the subtypes, if not, we're adding extra confusion at a regulatory level.
- I think below-species rank is definitely necessary but a big communication effort (outside of virology communities) should be done to enhance adoption and standardization
- As long as it is not mandatory for all viruses, I do not see immediate problems with supporting some virus names that are more known than the species it represents, e.g., parvovirus B19 (B19V), minute virus of mice (MVM), canine parvovirus (CPV), human bocavirus 1 (HBoV1), cutavirus (CuV), TTV, SARS-CoV-2, HIV, poliovirus etc. I think it would be good if ICTV also gave the preferred abbreviations, like the ones above. (As an example, it is confusing if human parvovirus B19 is called HPV).
- Some below-species are controversial, so I expect that there will be some disagreement. Maybe the study groups deciding below species-level classification should involve a broader community to discuss specifics.
- Acceptance by the field could be limited if currently existing/accepted designations would be changed? Would need to be plenty of consultation and information released to inform the communities - both scientific and non-scientific.
- ICTV may be attacked by claiming that it is not rightful to keep the right to name ALL virus classification level as it may be the democratic right of the individual reseerachers and experts. The SGs may be overwhelmed with this task so they just will not do it (as many of them are not proposing even the new species candidates for years). A possibility could be to enlarge the SGs according to the new needs.
- Regulations on biosafety may get still more challenging. Particularly, if one group/person is planning experiments to compare different isolates that may correspond to different below-species representants.
- It will be important for the study groups to engage and potentially align with other groups involved in the development of sub-species classification systems (outside if ICTV eg via disease specific networks or working groups etc).
- Taxonomy is expected to lag behind evolution in fast evolving RNA viruses during pandemics etc, might generate more confusion to officially classify variants at those times
- It will further confuse the distinction between viruses and virus species, and this would be exacerbated by the nomenclature that is proposed. Simply don't get involved in it. Specialist virologists already do and can in future use their own systems for classification of viruses below species level, subtypes, genotypes, clades, etc. In my experience, having spent many years working in a government biosecurity environment, few very scientists understand or even care about the difference between virus and virus species. Most scientists, clinicians and veterinarians think ICTV just keeps renaming viruses and feel very frustrated by that. This proposal would cause even more confusion.
- First, ICTV should help stakeholders understand the distinction between virus species and virus strains. The creation of sub-species ranks would be endless. I think that those should refer to taxa not to infectious entity.

- I simply prefer giving people in small communities studying a particular virus species the freedom to name and operationally classify stuff however they want below the species level. Maybe though rules would work well for bigger communities that are working on individual species.
- Probably need clear guidelines as to what constitutes a below species rank for each species so we don't end up with lots of "sub-species" and people using sub-species level as a way of increasing publication rates - e.g. publishing every single variant of a virus found
- We need to avoid changing names when not important to change since after several years the scientific community do not remember and there might have lost old publications
- Members of the study group are overall busy. I must say that it is already hard to accomplish the tasks attributed to the group in the present form. Introducing another taxonomic level will elevate the dedication time of the members, which will be a problem to keep them in the group. The below species level is complex, as for some viruses biological characteristics (host or symptom) are used, and for others, only the identity percentage of part (or entire) of the genome. Due to the wide variety of cases, it will make the members of the study group not confident to propose a defined rank, unless a careful study is undertaken. I do not support this idea, but I believe it can be useful for some particular viruses.
- What I'm fearing is that below species ranking may get overly complicated - especially when dealing with human/animal/plant pathogenic viruses - since pathogenicity may not be relevant to taxonomical classification. I'm curious to know how the medical virology community feel about lower taxonomical rankings.
- There's a well known joke about a tourist in Ireland who asks one of the locals for directions to Dublin. The Irishman replies: 'Well sir, if I were you, I wouldn't start from here'. It may be that the topic of below-species classification is in this category. I agree that other regulatory bodies have a problem with a few viruses of pathogenic importance, but I'm not convinced that it is primarily the ICTV's problem or that the best solution is to involve the ICTV in more work.
- You could approach the bodies concerned to see whether they would take on board an ICTV Executive member to advise, so that the kinds of errors illustrated in the proposal are avoided? Failing this, an ICTV guidance document on the small number of viruses concerned might straightened things out substantially.
- If the ICTV does become involved in creating subspecies, you should resist it becoming the thin end of the wedge, in which an option that applies to relatively few problematic viruses slowly invades the rest of the taxonomy. Operationally, a fast-track scheme that does not involve the full approval apparatus would be best.
- All this concerns formal taxonomy. I do not think that the ICTV should attempt to regulate the names of viruses or their variants.
- Provided the study group member for the affected virus families agree that subspecies classification is useful, and they apply naming conventions already in broad use, there should be no particular difficulties (except for folks who don't like change).The only difficulty will come with acceptance of the broader scientific and regulatory authorities if new or unusual subspecies classification names are introduced that aren't already broadly understood. For example, I note "clade" hasn't been mentioned in any discussion and this seems to be in regular use for influenza A viruses and some others. I'm sure the study groups are best placed to understand names in common use.

- The term "below-species rank" is somewhat arbitrary. A clear and consistent definition of such a rank and its divergence range (for instance subspecies or genotype or isolate) could be helpful.
- Communication and community involvement seem to be the keys in this endeavour. For instance, I am not sure how different parts of the community can feel represented if ICTV is the only body responsible for the below-species classifications. On the other hand, ICTV has been functioning as the main classification body since many years, so it is not only well-known across the community but also certainly established. All in all, ensuring that the interested parties are satisfied with the ICTV classification consensus & decisions is my only concern.
- The community still has to get used to the species binomial name. Introducing another taxonomic level, would then add another level of complexity. Per se, providing additional taxonomic resolution could be valuable, but I am concerned it is too early to implement this, at least in the plant virus community.
- Other point concerns the criteria for below-species classification; consideration of serological properties is valid, however probably limited by availability of the necessary serological tools, more commonly used in medical virology; genomic properties are another criterion, but the routine generation of genome sequences at high throughput is still developing. For these reasons, I see the introduction of a new taxonomic rank as more feasible in the future
- difficulties in defining demarcation criteria, various practices in categorization even between species of a genus, recombinants of subspecies, huge workload for study group members
- historical naming views, with solutions provided by virus species relevant genetic cut-offs
- It seems like there would be some resistance among some virologists to having to take an additional layer of classification into account.
- To me, as written in the proposal, the below species rank is written as a bit of a catch-all trying to do too much, rather than a useful classifier. This is highlighted in the following statement, “The advantages of clarity and flexibility provided through below-species assignments would similarly legitimize the introduction of essential virus names such as SARS-CoV-2 for the causative agent of COVID-19 by the ICTV Coronaviridae Study Group. (10). The existence of a below-species rank would enable the synonym, Sarbecovirus pandemicum isolate SARS coronavirus 2 to be provided.” The given example is also confusing as SARS coronavirus 2 is not an isolate but rather the broadest term for that strain. Additionally, there are widely used and clinically useful below strain classifiers used for the above example virus, such as delta, omicron, etc. for lineages and isolate names (e.g. WIV04). Below species ranks can become infinitely granular to individual unique nucleotide sequences, where do lines get drawn for useful classifications? Are all of these going to co-exist and be managed by the ICTV study groups? Will names end up like this, Sarbecovirus pandemicum isolate SARS coronavirus 2 Alpha WIV04? How will non-experts be able to tell the difference between all of these below species ranks? I agree that if implemented it must be limited and widely accepted by experts in the field and not just those that are members of the study groups.
- maintaining up to date classifications would be a problem for some viruses
- To complexify any taxonomy classificaiton its against clarity for non specialists (trust), visibility, ...

- Most new viruses are not published by the ICTV, and unless the new virus is a distinct species, there really isn't a mechanism for the committee to clarify species boundaries. This proposal fixes that. There will be some clean-up, but I don't see a problem.
- The potential friction going forward would be the potential for conflict in sub-species naming between the WHO and ICTV - between "variants of concern" and genuine taxonomy. In other words, we name viruses, except when virus names really matter. Then public health steps in and does the naming. It doesn't need to be a one or the other situation. This proposal broadens our remit from species naming to virus naming, and I think there is an important role for properly made phylogenetic trees and molecular understanding in virus naming.
- What this proposal does, is move virus-naming expertise a step closer to the public health response. We have been working better with the WHO lately, in my opinion. This move may not be without conflict, but I don't think conflict is inevitable. The benefits outweigh the risks, for me.
- Increased workload to uncertain benefits; I think that below-species classification should be encouraged only for certain groups of viruses, important medically or economically, not in general.
- It is likely that the below-species rankings would be very uneven among groups and depend on the presence of an expert (often a single expert) in the particular species in the Study Group. The info on this evolves very fast, and keeping pace with this would be difficult. Keeping obsolete information/classifications in the web page might be worse than having nothing.
- for biosecurity reasons wide consultations and consensus should take place for certain group of phages infecting pathogens with lysogenic characteristics which may result in the specialized transductions.
- More work for community, but worthwhile.
- ICTV has dealt with taxonomy up to the species level and made it very clear that naming of viruses are not part of ICTV. Including a below-species taxonomy would exactly adress the issue of different virus strains that have e.g. different serological reactions or pathogenicity on different hosts. The introduction of binominal nomenclature has already caused tremendious confusion in the plant virus world for diagnosticians, regulators and many others, I cannot see any benefit apart from creating more unnecessary work for our study groups
- While some below-species classification could be interesting for traits that distinguish some subgroups both biologically and molecularly (e.g., a molecularly distinct subclade of a virus with a distinct host range). That said, it will be difficult to find a "simple rule" that covers "everything". A distinction taking into account the virus-host biology would be relevant, but some biological properties depend on one or a few mutations, even a single amino acid can affect symptomology, transmission, or even host range.
- Existence of several, region-driven subspecies ranks/names and reluctance to accept the one defined by ICTV.
- Criteria for determining sub-species must reach significant consensus across groups working with these species and sub-species variants; process for establishing criteria would need to be transparent and inclusive
- I think one of the main challenges for sub-species classification lies in the phenotypic characterisation of viruses. For pathogens that evolve antigenically, such as influenza and SARS-CoV-2, even a single mutation can drive meaningful phenotypic change. Making these classifications systematic is difficult because it often relies on experimental validation, which is time-consuming and can yield variable results depending on the assay or experimental setup. In this context, machine-learning

approaches trained on existing antigenic mapping datasets could be a valuable complement to experimental work, helping to identify consistent patterns and guide more robust sub-species definitions.

- Downsides would be the time & discussions required to formalize these usages for each species. However if these are adopted per genus/species on an as-needed basis, then those groups who most want to employ these will be happy to put in the time to make it work. Their progress will show the path for those who follow.
- In plant viruses, the issue of quarantine pest lists in each country becomes messy.
- Backlash - many groups/people already involved in this area for their viruses - what you propose is the ICTV takes "ownership" of this and replaces them and their work - do you have the authority for that? (2) Speed - the ICTV is relatively slow, the taxonomy is updated once a year, and some parts of not being kept up-to-date - can the ICTV actually do this and quickly? Wont it look bad if this is adopted but only some viral species use it? (3) Would there suddenly be an standardised naming convention for genotypes etc that every virus would have to adopt?
- With subspecies classifications, Groups for viruses that don't need subspecies classifications may feel a need to create subspecies classifications. 2. Initially there may be confusion about this classification among biologists and the medical community at large. 3. This may cause issues with journals and some reviewers understanding and correctly editing viral taxonomy for viruses with subspecies classifications. 4. Education is obviously needed. The problem is, with the reach of this education. For example, it is obvious that some journal reviewers don't know the viral taxonomy for some of the papers they review, especially if they decide to review papers for viruses they don't regularly research. The breadth of such education and saturation into publication houses.
- Need for convergence on the below-species rank - need to organize the decisional process
- Issues: increased work load in processing proposals, documenting virus ranks/types, updating rank data in the public domain, dealing with complaints/disagreements, and potential increased legal liability (e.g. regarding regulatory authorities or phage therapy trials or litigation).
- Prevent/resolve: Provide very clear guidelines for proposals, and for study group members, so that the process does not lead to over-work and added stress for all concerned. Get legal advice to ensure there are no increased risks of litigation.
- The additional regulations and rules of naming subspecies/below-species ranks should be elaborated
- I think there are no consensus under-species classification scheme among different viral families. Some viruses diverge quickly, some do not; some has been classified by serological assays, some solely by genomics data. I believe threshold of different "genotypes", "serotypes" are also different among virus species. I agree that the best is to follow the current classification system for each virus species but it may result in classification of viruses at different divergence levels by families/species. At the same time, this could be a great opportunity to learn how other viruses in different species are classified into genotypes/serotypes.
- disagreement about details, resolve by well defined rules
- it might become very messy without clear, short, guidlines.
- The development of a complex naming scheme below the species level is likely to confuse the virology community. Already the continuous changes in virus species names is irritating, if the ICTV then starts interferring with names below species, it will be pandemonium.

- several classification (Eg enteroviruses) are not uniform , but yet widely excepted - eg specific types are assigned a certain way while other another way). Changing to a uniform classification might give issues
- lots of work for / lack of availability of SGs to study the below-species ranking proposals
- Because of different opinions about naming strains or isolates and therefore the recommendations for it should be elaborated by Study groups.
- Understanding, consistency
- this may slow down the process with more groups involved
- I can only speak specifically to bacterial viruses, as that is the area I work with. For these, I do not foresee more benefits in adding a subspecies level designation than downsides in the clutter and disorientation it would cause. Firstly, species nomenclature is already very challenging to enforce for bacterial viruses; terms like DAC 15, Crass001, and more overarching terms like crass-like phage are still used to this day in papers, presentations, and everyday usage. You will be hard-pressed to find many people who can recall the official names of some of the viruses they work with every day if these used to go by a common shorthand. If people wish to refer to a specific genome belonging to a phage, they will mention the gbk file or fasta file on an online database. While I believe that the addition of subspecies or strain designations to the ICTV wouldn't do much for bacterial viruses and would do more to slow down the taxonomy browser and add perceived complexity to the classification of viruses, a process some researchers already consider more hassle than duty. If these were to be added in any capacity, I would rather have the ICTV manage cutoffs and definitions for these entries than any other party. https://doi.org/10.1093/molbev/msaa314 is a paper that comes to mind regarding the confusion caused by a lack of guidelines for classifying subspecies. Having a committee or even a web tool to determine if a genome is fit to be considered a novel subspecies would significantly reduce taxonomic bloat.
- To summarize, I don't see the need to store subspecies entries in the ICTV (mainly for bacterial viruses) as species level designations are not commonly used in communication, no subspecies designation will be clearer than simply giving the genome/accession (especially since, depending on the demarcation criteria set, subspecies might not tell you anything more than species designation) and since the ICTV requires all MSL members to be available on the NCBI, all possible subspecies designations would link back to an NCBI entry anyways.
- Either set a strict guideline on subspecies designations (99.5% total ANI or something similar https://doi.org/10.1128/mbio.01536-24) or make use of the fact that NCBI will separate genome entries of the same species and let the user decide what level of differentiation they are interested in.
- The introduction of a below-species rank is likely to exacerbate confusion within the virologist community, particularly regarding the distinction between virus name as a physical entity and the designation of species. Establishing general applicable demarcation criteria for below-species rank would present significant challenges. In addition, the inability to abbreviate subspecies names would further hinder clarity and efficiency in scientific writing.
- It may lead to below below species and below below below species classifications.
- Just like the concept of binomial taxonomy, it is important that everyone in the virology community understands the significance of this new change and what it entails. This can be achieved through webinars or training courses for people who works in the viral families affected.
- ICTV is already far from being up to date in the classification of viruses at other taxonomic rank (some taxa have a classification of high quality whereas others have not

been updated for a very long time and require very significant update). The community needs to strengthen its base, and demonstrate its ability to adequately classify viruses down to the species level. Moreover, the change of virus species names is not yet well accepted in the community and the ICTV should strongly restrain from renaiming viral strains. I think the ICTV can be used to record accepted strain names and classification but should restrain from driving re-naming of viral strains through study groups.

- Inconsistencies between groups of viruses. Also potentially categorising things as "taxonomy" that aren't necessarily reflective of evolutionary origins.
- Confusion among people not familiar of how viruses are named
- increased workload. Potential for unclear evidence - decisions regarding use of subspecies may not always be clear, so strict usage guidelines will be required
- Opposition from SGs that do not have to deal with below species classification
- It will mean more work for ICTV and its members
- The primary issue will be explaining a new level and it's value while making it clear that it will not be a default universal level for all viruses. This will require a consistent, clear message and a robust approach for formally accepting its application where appropriate. It will be important to keep 'control' of the new designation to avoid an explosion of potentially confusing and ill thought through new designations.
- Proliferation of efforts to describe strains or subspecies for plant viruses
- This might affect differently to each SGs
- variability in its application across viral groups
- Persistence of old nomenclature. There is a continuing challenge in reaching consensus and a persistence of alternative nomenclatures across publications. Another major challenge lies in establishing appropriate cut-off values. In some cases, sub-species were previously defined as distinct species; in other instances, what are now considered sub-species would correspond to the so-called genotypes.
- increased workload on ICTV study group members, longer delays to get anything classified even at other ranks
- Uselessness of species names will be clarified. To solve this problem, species names should be abandoned and common names should be used instead as before.
- I think it will require more time commitment initially to get everything up to date. I think it would be useful to have some firm of comment period from researchers to give feedback. I think this part will be very divisive and getting feedback from the larger community will be good.
- Taxonomic classification should follow evolutionary relationships (phylogeny), while current demande for creating sub-species level classification focoses on viral phenotypes. This contradiction should be addressed prior to the decision on making it or not.
- The usual problems reconciling older designations based on antigenic and other phenotypes with current data that are almost exclusively genomic
- Although most virologists are capable of performing adequate phylogenetic studies, below-species assignments determined primarily by virus biology will be beyond the scope of work performed by many of those identifying viruses from high throughput sequencing, and lacking facilities to carry out biological assays (host ranges, serological differentiation, etc.). This may result in many cases of virus isolates not being characterized to below-species level for species for which below-species level classification might exist. It presumably would not be required for isolates to be assigned to below-species level, even though such assignment might be useful.
- I expect that different fields of virology will have historical and other reasons for strongly preferring disparate sub-species nomenclatures.

- There may be confusion at the beginning of the introduction.
- The below species ranks would be challenging as there can be different characters for every virus. The study groups in plant virology already have a difficult job with species discovery.

2) *General comments on Section C (42 responses)*

- The proposal generates additional confusion between the species (abstraction) and the causative agent (the virus). It seems aimed at giving a taxonomic status to virus strains, serotypes, types..., which are not below species levels but are variants of viruses belonging to the same species. There are many examples in the proposal which are not correctly formulated. For instance, the sentence "...where the species term, Alphainfluenzavirus influenzae, would be similarly inadequate to distinguish the causative strain, eg. HPAI-H5N1, from seasonal influenza A viruses" is misleading.
- It was unclear if the below-species classification will be for example, strain, type or similar category.
- Consider:
    - ADVANTAGES
    - Adds Precision Where Species-Level Classification Falls Short
    - Formalizes Existing Community Classifications, giving them standardised, ICTV-approved names.
    - It is flexible for Study groups that can use it when helpful without forcing classification for all viruses within a species.
    - Aligns ICTV With Other Biological Taxonomy Systems in line with zoological/botanical codes that already have subspecies-level categories.
    - May help identifying/clarifying virus common names from binomial species names in databases and regulatory lists (WHO, ACDP, NCBI, etc.).
    - may improve use and clearer communication for clinical, veterinary, plant health, and regulatory authorities.
    - DISADVANTAGES
    - Increases Complexity of the Taxonomic System
    - There is a risk of inconsistent adoption because is it’s optional.
    - there is potential for Naming Disagreements
    - It may be not universally necessary for all species or new discovered species
    - Will create Extra Work for Study Groups by defining criteria, reviewing historical usage, and proposing below-species taxa
    - Benefits outweigh dangers, but the reform must be carefull, systematic and enforced with conviction.
- The genotypes of Hepatitis B virus (species: Orthohepadnavirus hominoidei) are already integrated into the current Genus: Hepadnavirus and are widely accepted in the field.
- The concept of subspecies already exists for many viruses. For example, earlier classifications of flaviviruses included subtypes. Therefore, an introduction and a clearer definition would be useful. It should be noted that previously, concepts such as genotype (differences were based solely on phylogenetic analysis), serotype (differences were determined by serological reactions, primarily the neutralization reaction), and subtype (the coincidence of genetic and serological differences) existed. Clearly, a very clear definition of subspecies will be needed to avoid misunderstandings.
- I would favour including sub-specific categories in the VMR as an option rather than a requirement. Similarly, ICTV Study Groups should be encouraged to develop sub-specific categories where there is a need for this, but not where such categories have already been developed. So complement what is already being done rather than formalise it.

- I believe that any classifications below the species level that the ICTV supports should be those agreed upon by experts and published, as well as supported by international institutions such as the WHO. This would avoid conflicts and be very helpful when selecting the classification to be used for genomic surveillance. Examples of such consensus classifications include RSV (doi: 10.3201/eid3008.240209), SARS-CoV-2 (doi: 10.1038/s41564-020-0770-5), dengue (DOI: 10.1371/journal.pbio.3002834), etc.
- My primary concern is that this proposal addresses a virus nomenclature problem by expanding taxonomic classification, which may not be the appropriate solution. The ICTV's mandate is to classify virus species as taxonomic units, not to formally name or classify individual viruses, strains, or variants that exist within those species.
- For the virus families I work with (Endornaviridae and Hadakaviridae, as Chair and member respectively), there is no practical need for below-species classification. These viruses lack infectivity, cannot be experimentally transmitted, and are classified based on sequence similarity. The current species-level framework provides sufficient resolution.
- I recognize that needs may differ for other virus groups, particularly medically important viruses where regulatory and clinical distinctions are critical. However, these needs might be better addressed through mechanisms outside of formal ICTV taxonomy, allowing the community to develop practical nomenclature systems while ICTV maintains its focus on species-level classification.
- If below-species ranks are adopted, I believe reviewing and refining species demarcation criteria should be prioritized, as this addresses a more fundamental issue in virus taxonomy.
- Even not official, the strain or subtype levels are used by scientists
- Below species classification will greatly help to classify viruses properly, as it will also provide an accurate framework for GenBank and solve multiple cases of ambiguous taxonomy or wrong entry labeling.
- After so many years working on unifying taxonomomy and consolidating species lists, it seems a step back
- I recommend a gradual progression toward implementing below-species rank classifications, as this approach is likely most suitable for important or well-characterized viruses at the current stage.
- Such an additional sub-classification should add additional limitations to the reserach of those viruses.
- The use of additional taxonomic sub-divisions is potentially endless. If further granularity is really required to resolve viruses with sufficiently different properties, they should simply be classified to different virus species by setting appropriate demarcation criteria.
- I think its probably a good idea especially for the medical community. It could also be helpful for viruses that have small portions of their genome recombine, but not enough to call them a new virus species. - E.G TUYV
- If below species rank will be established by ICTV the information should also be easily found from the ICTV webpages (e.g. the Master Species List).
- Clarity on questions posed in this section would improve through holding on-line conference on the topic that will explore contrasting perspectives; better before making decision. Some extra issues: a) feasibility of reliable genome-based classification of clusters below species; b) advantage(s) and/or added knowledge compared with the current ad hoc system; c) share of virus species that may benefit from this classification; d) interaction of ICTV with other bodies that may be concerned and possibly be responsible for this activity; e) ...

- below species classification affected by genetic drift and replication fidelity per virus species
- In case of Solemoviridae members, there is a need for the subspecies rank for natural recombinants that multiply and spread (sometimes in different geographic regions, or even having differences in their host range) but are not sufficiently different in their full genome or RdRP sequence level.
- It's a good idea to include the information in some capacity
- below-species classification of the viruses needs to be SPECIFIC for each virus family (in reference to the criteria: BIolofy, phenotype, genotype, phylogeny etc...) No general rule
- Might be better to discuss within a workgroup to better understand how it might apply to poxviruses
- Previously, the ICTV correctly limited its classification work to the species level. Sub-species classification working groups—formed with or without ICTV members—provide greater professional representation for the relevant viruses; therefore, I do not see a need for the ICTV to address this separately.
- I am the chair of the Betaflexiviridae (plant viruses). Many viruses are assigned solely based on genome sequences, and many have little or no biological characterization. It is not adequate to officially establish a subspecies rank within each species in this family.
- Just a thought if instead of creating subspecies level bin, there should be an effort made to change species demarcation criteria to move the subspecies taxa to species level, where those are defined within clear monophyletic lineages
- See my comments on previous section.
- Phages are so diverse that even the species level is barely useful. I reason at the genus level.
- I think this is a brilliant idea because the virus strains and genotypes are specific designations of virus entities that can be traced and confirm the authenticity of a virus preparation or virus sample.
- All sub-species classifications should be clades. Phylogenetic trees on whole genomes should be the main guide, not clinical similarity or serological criteria.
- The current justification for below species classification seems reasonable.
- We don't want to lose information on metagenomic viruses which do not fit the current ICTV systematics
- I understand why this has been proposed, I'm just not sure if it's actually helpful or not. Virus-specific classifications can still exist alongside taxonomy (I know that some people seem to struggle with this). HIV-1 is a virus, and it belongs to the species Lentivirus humimdef1. Taxons can still exist alongside virus names....
- I understand the significance and necessity of classification below the species level, but there remains doubt about whether such classification should be adopted for all viruses.
- In fact, some researchers around me have stopped considering classification altogether (ignoring it) due to the shift to binomial nomenclature. Even if the change feels natural to those creating the classification, it is difficult to ensure all researchers understand the rationale. Therefore, I believe changes to complex classifications should be avoided.
- Furthermore, the necessity of sub-species classification varies for each viral species. Therefore, while adopting classification should follow the proposal, I feel that whether to adopt sub-species classification at all requires discussion within the Study Group.
- ICTV review of major subspecies designations would be helpful, while naming of transient variants or specific genetic mutants may be best left to academic groups
- Below species rank should formalize criteria for current genotypes in paramyxovirus species that is bound to expand with metagenomics studies

- I would be in favor of ICTV recording community usage and consensus for below-species classification, but without any role for a study group or the EC in terms of establishing names, convention, etc. Essentially adding this information as part of the MSL to better connect these below-species rank classifications used in the literature and the community to the above-species (and species) classification established by ICTV.
- For the regulatory affairs, only human pathogenesis/intervention possibilities matter. However, this rarely matches with subspecies ranking.
- I think that the species names are making confusions and unnecessary.
- Whilst some clarity, such as a position statement on the importance of biological characteristics in determining strain, as opposed to genetic clade, would be welcomed, the ICTV have always argued that below species ranks are not their preserve as that gets into the "virus" not the "species" descriptions, and as such could be considered to be mission creep.
- I just think that a homogeneous naming of viral isolates could perhaps help

Section D. Format of below-species nomenclature

General comments on nomenclature and name formatting (55 responses)

- I worry that ICTV species designations and nomenclature have already diverged from the practices and language of virologists and that imposition of additional rules and new nomenclature would just make that worse.
- The ICTV may provide recommendations for writing such designations — not as part of formal nomenclature, but as stylistic or technical guidelines. In this way, the ICTV would enable a consistent system of notation in publications and databases, without introducing new taxonomic ranks.
- It will not be possible to formalize a below-species nomenclature. Again, it will follow clinical practice which concerns virus names
- No more than a single unified designator.
- Italicized epithets should be used for subspecies. Not for serotypes or strains.
- If sub-specific ranks are to be formally recognised, the format should clearly distinguish them from non-formal designations
- We should try to make it as easier for the transition as possible. And as clear as possible too.
- Regarding the question: Italicise below-species rank names? It becomes complicated at some points to describe what a virus "does" if the name is not precise enough taking into account the taxon do not "do" anything: they do not replicate, they are not transmitted,...
- The way of naming each virus below its species should not be generalized, but rather that approved by the consensuses that I have been discussing in the previous answers.
- It makes absolutely no sense to Latinise the naming format below-species rank, if the species epithet is not Latinised first. I would always Latinise the species epithet first, and keep alphanumerical codes for the below-species rank names.
- As I do not believe ICTV should extend its mandate to include below-species classification, questions about the specific format and nomenclature of such classifications are not relevant to my position. These details of virus nomenclature would be better addressed by the virology community and relevant stakeholders outside of the formal ICTV taxonomic framework.

- Keep things simple. Use old names for continuity and a single designation ssp. I already spend enough time translating virus nomenclature to regulators. A single clear, consistent and traceable approach please.
- I meant above that it is fine for below-species viruses to have genotypes or serotypes, which well can have a "type" number (e.g. parvovirus B19 genotypes 1-3 and bufavirus 1-3), without combining them with the species name
- I strongly support this proposal :-)
- In the case of adenoviruses, type would practically mean serotype, genotype could mean a recombinant (have having the same serotype/hexon as an earlier described "type")
- Genotype or subspecies designator
- Just don't do it.
- subspecies or range of designators
- As commented previously, I am uncertain whether the ICTV should create and regulate subspecies taxonomy, but I have answered the questions on the basis that it decides to do so. I think a subspecies name should be italicised, as the whole construct is a taxonomical name and not a virus name, and I do not think the ICTV should seem to name viruses.
- Thank you for seeking the opinion of all ICTV Study Group members.
- ICTV does not have enforcement power and must rely on public/field support (of below species classification) to see new names used and as intended.
- A unique solution applied throughout the whole virus taxonomy is probably not very possible, so I would opt for a range of designators below the species level. Italic fonts and latin language are probably not a good idea, since many are annoyed by these two (or don't know how to use them, or both).
- Established designators are used, but they are not coherent within the different species of the genus.
- Make clear is a subspecies and also that this is a dynamic category with further data could be species level
- should be relevant to what is currently used
- Altogether, its time consuming and $ and mots of the virus family has already its ownw rules (type, subtype, genotype, serotype ....)
- No need to discuss these questions unless the community of ICTV agrees to deal with classification below species level
- I'd prefer to stick to Latinized nomenclature, but base it on existing familiar names as much as possible.
- For formalized classification, I think it would be more fitting to mandate the use of a single subspecies designator (such as ssp.) across the entire virus taxonomy for consistency.
- Do not regulate below-species classification at all and definately not in this Latin bullshit!
- "Each species and each family has its own circumstances. It is not adequate to establish a general rule for subspecies classification for all study groups.
- I dont think the species name should appear anywhere - just the subspecies element e.g. serotype SAT2, SAT2, genotype Asia1, Asia2, lineage Alpha, Beta etc. I dont think there should be any restrictions on naming here, the species name is the key - these are subpopulations of that species. A suggestion would be a case by case situation. If an ICTV study group wants to create a new sub-species classification system then this should be presented in a proposal. If the ICTV study group is the de facto authority in this area already - then great. However, if one already exists it needs to be shown that this is significantly problematic and that the user community wants to replace it. Seems a bit

odd that the ICTV can suddenly claim ownership of this across the viro-sphere leading to lots of publications for them, and ruling out everyone else who already works in this area. Will all study groups be expected to come up with rules for sub-species classifications?

- I think the decisions about a third taxonomic name or subspecies designator should be left to those most affected by such changes.
- Definitely include virus name, e.g. poliovirus, but different virus-host systems use very different terms, for example influenza subtypes are named very differently from plant virus strains/isolates.
- Keep Letters for species and numbers for sero-/genotypes
- This is an excellent proposal that should be implemented ASAP.
- The main reason for my "No" to designators is that there may be a proliferation of them. If we have to use a designator, it should be formalised, such as "sub-species", e.g. Enterovirus coxsackiepol subsp. poliovirus 1
- This should ideally be the NCBI accession or the last few characters of the accession; it would allow people to instantly find the strain without adding much confusion. While this would be a bit awkward to pronounce, it would heavily cut down on the amount of extra searching that a change like this would add.
- The issue of nomenclature and typography at the below-species rank could constitute a significant obstacle in the implementation of the proposed new level of classification. In my view, none of the aforementioned options appear to be feasible. The establishing of demarcation criteria for below-species rank, coupled with the complexities of nomenclature and typography at this classification level, risks undermining clarity and consistency in taxonomic practice. Moreover, the absence of standardized abbreviations for subspecies names would impose additional burdens on scientific communication, complicating both written discourse and database management.
- This proposal has not been discussed in our ICTV study group.
- If new (metagenomic) viruses do not fit the current taxonomic structure they should be identified by an easily recognised structure, say italics
- Publication with bionomial species name without virus name and abbreviation confuse readers.
- It is very much necessary to make it as easy as possible for everyone already studying these viruses to NOT feel that there has been any change in their practice. Additional modifications can still be implemented later on once these first set of chanegs are accepted.
- My comment on the previous page was probably more relevant here.
- While the use of current designators will result in different designators for different species which some might find troubling it will emphasise the nature of the designator for each virus. This will better reflect the reason for the designation, which is appropriate for its intended justification as indicated in the initial comments for this exercise, and will make it easier for those working in areas where a specific attribute is relevant for their area of interest. For example, for a researcher with an interest in virus antigenic changes a designator of serotype would allow them to seek out examples from a wide range of virus families with comparative ease. If a single designator was used ghis selection would not be possible.
- In my view, it would be most helpful to include existing widely used nomenclature to avoid confusion and to keep researchers adherent to ICTV. I think it would be chance to further align currently used virus names with formal binomial ICTV nomenclature.
- it might get too complicated to designate differently all type of subspecies designators, using ssp for all might simplify that

- I think that this problem indicates that the rank "species" was unnecessary for viruses.
- Not quite sure about the above question. It depends on how many layers you want to go. If you are going to keep traditional virus names and then break into genotype that's 2 additional layers below species. I don't think this is a bad idea and would solve the confusion issue with virus names and put the phylogeny in one place. Just not sure how I would label those levels. The virus name isn't really a subspecies, so maybe just add column headers appropriate for the data, common name, genotype...
- We have to distinguish taxonomic classification (evolutionary relationships) and practical classification (e.g., serotypes, etc.) The latter should not be the basis of sub-species classification in principle.
- Some flexibility in format is needed to avoid major confusion when referring to the older literature
- Existing below-species designations for host or sequevar genotypes of turnip mosaic potyvirus would not translate to latinization in a meaningful way; however latinization of wholly new below-species differentiations might be useful
- Where is the clarity between the species and the virus nomenclature in the above examples - surely isolate, type etc refer to the virus, not the species? Within plant virology there are some phylogenetic splits can be made, however, these do not clearly link ot biological traits, and as such giving formal descriptors could lead to regulatory challenges.
- Add the same third taxonomic name to the vernacular name of the virus
- Example - Genus species sub-species - Gammanudivirus cracrangonis cracrangonis
